# Real-Time HD Map Change Detection for Crowdsourcing Update Based on Mid-to-High-End Sensors

**DOI:** 10.3390/s21072477

**Published:** 2021-04-02

**Authors:** Pan Zhang, Mingming Zhang, Jingnan Liu

**Affiliations:** 1 School of Geodesy and Geomatics, Wuhan University, Wuhan 430072, China; panz@whu.edu.cn; 2 Gui Zhou Kuandeng Zhiyun Science and Technology Ltd., Beijing Branch, Beijing 100016, China; zhangmingming@kuandeng.com

**Keywords:** HD map, crowdsourcing update, semantic segmentation, visual SLAM, autonomous driving

## Abstract

Continuous maintenance and real-time update of high-definition (HD) maps is a big challenge. With the development of autonomous driving, more and more vehicles are equipped with a variety of advanced sensors and a powerful computing platform. Based on mid-to-high-end sensors including an industry camera, a high-end Global Navigation Satellite System (GNSS)/Inertial Measurement Unit (IMU), and an onboard computing platform, a real-time HD map change detection method for crowdsourcing update is proposed in this paper. First, a mature commercial integrated navigation product is directly used to achieve a self-positioning accuracy of 20 cm on average. Second, an improved network based on BiSeNet is utilized for real-time semantic segmentation. It achieves the result of 83.9% IOU (Intersection over Union) on Nvidia Pegasus at 31 FPS. Third, a visual Simultaneous Localization and Mapping (SLAM) associated with pixel type information is performed to obtain the semantic point cloud data of features such as lane dividers, road markings, and other static objects. Finally, the semantic point cloud data is vectorized after denoising and clustering, and the results are matched with a pre-constructed HD map to confirm map elements that have not changed and generate new elements when appearing. The experiment conducted in Beijing shows that the method proposed is effective for crowdsourcing update of HD maps.

## 1. Introduction

Autonomous vehicles use various sensors to achieve different levels of autonomy (L1-L5, e.g., see [1]), such as cameras, Global Navigation Satellite System (GNSS), Radio Detection and Ranging (RADAR), Light Detection and Ranging (LIDAR). However, these sensors have a limited perception range, and they are very vulnerable to bad weather. To overcome the limitations, the pre-built digital map can be utilized to improve perception and robustness. Many autonomous vehicle prototypes rely on precise 3D maps [2,3], which are also called high-definition (HD) maps. An HD map is a precise map with rich lane-level information for autonomous driving. It can provide prior information robustly about the static environment in a range of more than 200 m ahead or around corners. The features in the map can be fused with the recognition results from camera/LIDAR to realize high accuracy localization of the vehicle [4].

Compared with the car navigation map, an HD map greatly improves the accuracy to a few centimeters level [5]. It also has richer and more detailed content, such as lane boundaries, lane centerlines, road markings on the ground, and guardrails on both sides of the road. Lane boundaries have many attributes in HD maps, such as type, color, and width. Therefore, HD maps reflect a more realistic and detailed real world, containing a lot of subtle changes. For example, the lane boundary is re-brushed, and arrows are added to the road surface.

At present, the production of HD maps requires professional data collection, that is professional surveying and mapping using the Mobile Mapping System (MMS) [6,7]. Then, HD maps are constructed from the road images and 3D point cloud data. The entire data collection and production takes a long time. In addition, the professional survey fleet is very expensive to set up. All of these make it difficult to update the HD map in real-time.

Therefore, more and more researchers focus on crowdsourcing update of HD maps. In the future, driverless cars will be no different from professional survey cars as they are equipped with similar sensors. So, when they are driving, they will also be collecting data. The collected data of every car can be aggregated and then used to update HD maps. This is the concept of crowdsourcing updates of HD maps. Once the map update is completed in the cloud, the update package can be passed back to the vehicles. Then real-time HD map updates and services can be realized.

Ref [8] uses a single front-facing camera, a consumer-grade GNSS/IMU, a Qualcomm Snapdragon 820A SoC in the vehicle, and a backend mapping server to realize the crowdsourcing update of traffic signs and lane boundaries. Every single-journey perception data and triangulation outputs are shipped over a commercial LTE link to the backend mapping server. Thus, the amount of data transmitted by the network is very large. [9] proposed a generation method of new feature layers in the accuracy level of the HD map using the existing HD map and crowd-sourced information without additional costs. The generated new feature layer is uploaded to the map cloud by the mobile network. The amount of data transmitted is small, but the computing power requirements on the terminal are high. In addition, it focuses on the new feature types that have not appeared on the HD map.

Therefore, there are many different strategies based on different sensors and processing methods for the crowdsourcing update solution.

- First, what kind of sensors are there in the car, and what is the accuracy range of these sensors? For example, is the LIDAR included, and what is the accuracy of GNSS?
- Second, whether the full amount of sensing raw data is uploaded to the cloud, only some keyframe data is uploaded, or just the recognized results are uploaded.
- Third, is there an HD map on the end? If yes, whether the difference data between the HD map and real-time environment perception needs to be uploaded.

There are no very definite answers to these questions. However, there is no doubt that a reasonable sensor configuration and the corresponding processing method are very important for large-scale crowdsourcing update in order to ensure efficiency and reduce the amount of transmission data.

Currently, most social vehicles are not equipped with advanced sensors, only cameras for dashcam and low-precision positioning system for navigation. There is no strong perceptual computing power to process the original image. However, in recent years, mass-produced cars with automatic driving above L2 level, such as Audi A8 and BMW iNext, are not only equipped with cameras, millimeter-wave radar, and other sensors, but also equipped with a high-precision positioning system, HD maps, as well as chips or domain controllers with powerful real-time sensing computing power. Therefore, crowdsourcing map updating based on mid-high-end sensors is becoming more feasible and will be the trend in the future. In this paper, based on these mid-high-end sensors, vectorized real-time perception data is generated after real-time semantic segmentation, SLAM and other key technical processing. Then through the matching between the vectorized data and the pre-constructed HD map, map elements that have not changed are confirmed and new elements that are not on the map but in the real world are generated. This also means that real-time HD map change detection is realized for crowdsourcing update.

## 2. The Architecture

**Figure 1:**
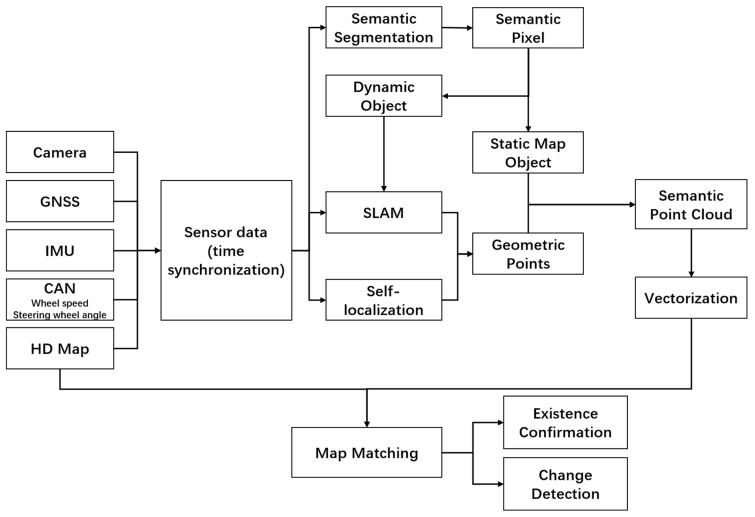
The architecture.

This paper proposes a real-time HD map change detection method in the vehicle terminal based on an industry camera, a high-end GNSS/IMU, and a high-performance onboard computing platform. Semantic SLAM (Simultaneous Localization and Mapping) technology is mainly used to obtain semantic point cloud data of lanes and other features based on an improved BiSeNet network [10]. Then, the semantic point data is vectorized and matched with the HD map to detect differences. The proposed architecture is shown in Figure 1.

According to the architecture, the system comprises several modules as follows.

- Localization. Localization is one of the major subsystems of autonomous vehicles. Currently, the main sensors used for localization on vehicles are GNSS, IMU, cameras, and Controller Area Network Bus (CAN Bus) information such as wheel odometer. GNSS and IMU are commonly used to form an inertial navigation system. In this paper, NovAtel SPAN-IGM-A1 [11], a commercial integrated navigation product, is directly used to simulate the high-end positioning system on the vehicle. When using RTK (real-time kinematic) mode, it can provide a self -positioning accuracy of 20 cm on average. In this paper, we do not address this module.
- Image semantic segmentation. Semantic segmentation amounts to assign semantic labels to each pixel. With the development of deep learning, some networks could achieve good performance in semantic segmentation [12]. BiSeNet is one of the best real-time semantic segmentation networks in recent years. Thus, it is chosen to recognize the images collected by a single front-facing camera. In fact, this paper optimizes the BiSeNet network to improve recognition performance. NVIDIA DRIVE AGX Pegasus™ is utilized to simulate the onboard high-performance computing platform. Static features such as lane boundaries, road markings, traffic signs, and moving objects such as vehicles can be recognized with a relative high IOU (Intersection over Union). IOU is a commonly used measure for determining how accurate a proposed image segmentation is, compared to a ground-truth segmentation. Section 3 covers the image semantic segmentation in detail.
- Semantic visual SLAM. SLAM aims to self-localize a robot and estimate a model of its environment from sensory information [13]. The framework of the visual SLAM system is quite mature, which is generally composed of several essential parts such as front-end, back-end, and loop closure detection [14]. Some advanced SLAM algorithms have already attained satisfactory performance, such as feature-based ORB-SLAM2 [15], direct method LSD-SLAM [16], and semi-direct visual odometer method (SVO) [17]. Vision SLAM can produce geometric maps composed of points or edges but without any associated meaning or semantic content [18]. As every pixel in the image has a known type after semantic segmentation, the usual SLAM maps can be enriched by associating the geometric estimation with object information. This process is called semantic SLAM [19]. In addition, the adaptability of visual SLAM to the dynamic scene is generally poor because of the limitations of the sparse image features. The constructed map often contains moving objects. Due to the influence of moving objects, there will be residual shadows of moving objects on the map. The semantic segmentation results can be used to remove these moving objects in the dynamic scenes to improve the quality of SLAM maps. Through the implementation of semantic visual SLAM technology, we can get semantic point cloud data with the spatial location of features such as lane boundaries, road markings. The details of semantic visual SLAM are described in Section 4.
- Vectorization and HD map matching. The semantic point cloud data is vectorized after denoising and clustering. The KD-tree and RANSAC algorithms are used. Then these vectorization results are matched with the local HD map to detect changes. The details of vectorization and HD map matching are given in Section 5.

## 3. Semantic Segmentation

NVIDIA DRIVE AGX Pegasus™ is used to simulate the vehicle-mounted high-performance computing platform. It achieves a 320 TOPS (Tera Operations Per Second) of supercomputer. Its next generation product will increase the computing power several times [20]. Therefore, it is foreseeable that the on-board computing power will continue to increase. This also means that more and more processing can be done on the end, such as images semantic segmentation, object detection, and so on.

BiSeNet [10] is a real-time semantic segmentation network proposed by Megvii Technology on ECCV2018. Using Res18 as a base model, the fast version of BiSeNet achieved the result of 74.8% Mean IOU (mIOU) on the CitiScapes verification dataset at 65.5 FPS. Our application scenarios require far less real-time performance of semantic segmentation than 60 fps, so we can improve the network to reduce the real-time performance, but increase the segmentation accuracy.

**Figure 2:**
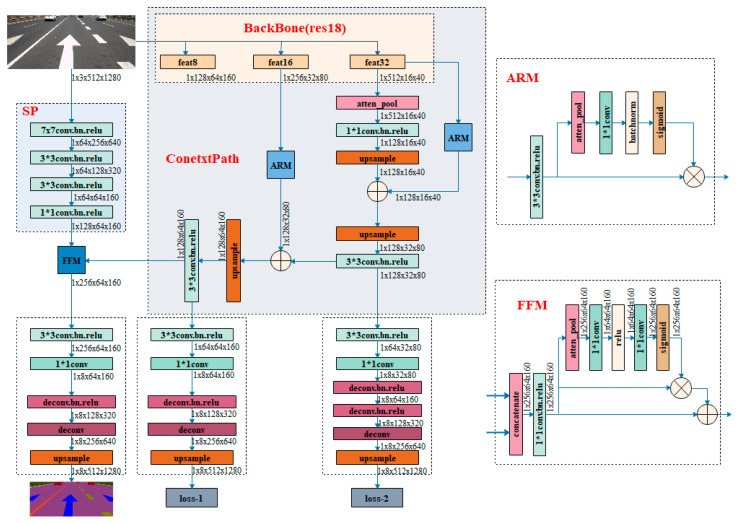
The improved network based on BiSeNet.

The original BiSeNet consists of two parts: Spatial Path (SP) and Context Path (CP). These two components are used to confront with the loss of spatial information and shrinkage of the receptive field. Spatial Path is designed to retain the spatial information of the original image. Context Path utilizes the lightweight model and global average pooling to quickly obtain a large receptive field. We have made targeted optimizations to these two parts. The improved network architecture is shown in Figure 2. The details of the improvement are as follows.

(1) The original network uses the method of upsampling 8 times, 8 times, and 16 times in the last three output layers to directly restore the original size. It is modified to restore the image size by 4 times, 4 times, and 8 times through deconvolution. Finally, the original image size is restored by 2 times upsampling directly.
(2) The classic attention idea is used in the original network, that is, average global pooling is utilized to obtain a sizeable receptive field. After our optimization, local attention and multi-scale attention are used to further improve the segmentation performance.

The modifications are shown in Figure 2, such as the abbreviation “deconv” for deconvolution. The key point is that we use deconvolution for restoring the original size of the image. The realization of deconvolution needs to obtain parameters through learning, so as to achieve higher accuracy. The original BiSeNet uses interpolation for upsampling directly. The advantage is that it is fast and does not require to obtain parameters. The disadvantage is that the accuracy is lower than deconvolution.

Regarding the loss function, the original BiSeNet uses Softmax loss and we adopt focal-loss [21]. Softmax loss is essentially a kind of cross-entropy loss function [22]. Focal-loss adds weight on the basis of cross-entropy and solves the problem of sample imbalance. So focal-loss performs better in accuracy.

The first step of focal-loss is the cross-entropy loss function for binary classification, defined as:

(1) $$CE\left(p,y\right)=\{\begin{array}{c} -log\left(p\right)\;\;\;\;\;\;\;if\;y=1\\ -log\left(1-p\right)\;\;\;\;otherwise. \end{array}$$

In the above, y∈{−1, 1} specifies the ground-truth class and p∈[0,1] indicates the model’s estimated probability for the class with label y=1. For simplicity, we define $p_{t}$:

(2) $$p_{t}=\{\begin{array}{c} p\;\;\;\;\;\;\;if\;y=1\\ 1-p\;\;\;\;otherwise, \end{array}$$

Thus,

(3) $$CE\left(p,y\right)=CE\left(p_{t}\right)=-log\left(p_{t}\right)\;\;$$

Then in order to address class imbalance, a weighting factor α∈[0,1] is introduced:

(4) $$\alpha_{t}=\{\begin{array}{c} \alpha \;\;\;\;\;\;\;if\;y=1\\ 1-\alpha \;\;\;\;otherwise, \end{array}$$

For simplicity, we define $\alpha_{t}$ like we defined $p_{t}$.

(5) $$CE\left(p_{t}\right)=-\alpha_{t}log\left(p_{t}\right)\;\;$$

Then a modulating factor $\left(1-p_{t}\right){\;}^{\gamma }$ is added to the cross-entropy loss for reducing the loss contribution from easily classified samples: γ≥0, which is a tunable focusing parameter.

(6) $$m=\left(1-p_{t}\right){\;}^{\gamma }\;$$

Thus, the focal loss is defined as:

(7) $$FL\left(p_{t}\right)=-\alpha_{t}\left(1-p_{t}\right){\;}^{\gamma }\;log\left(p_{t}\right)\;\;$$

**Figure 3:**
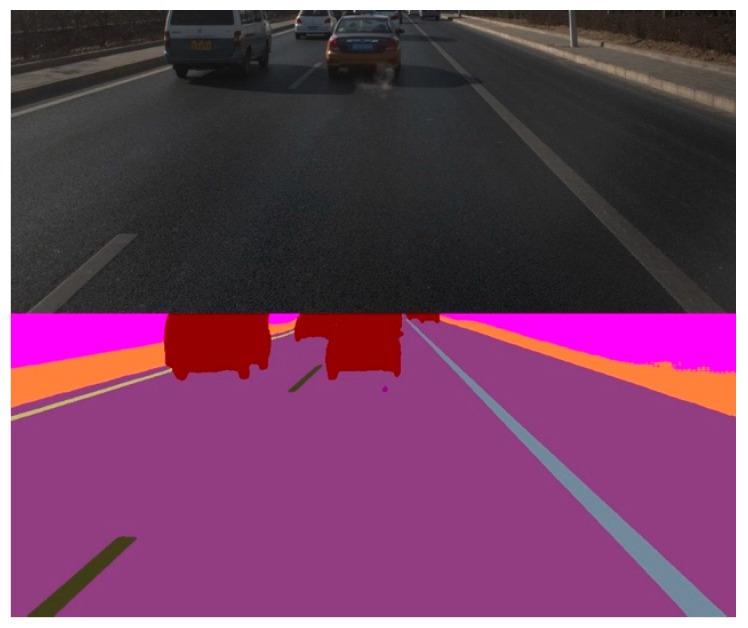
An example of image semantic segmentation.

The data set for model training and verification comes from the image data collected by Kuandeng Technology. The original image resolution is 2048 × 2448, and there are more than 80 types of labeling categories. The data set has a total of 11,830 images, including 10,597 images in the training set and 1240 images in the verification set. The recognition results of the main categories are shown in the Table 1. The average IOU reaches 83.90%. While the average IOU of the original BiSeNet is 76.8%, which is very close to its results on the CitiScapes dataset. Thus, after our optimization, the accuracy has been improved. The IOU of objects on the road such as vehicles reaches 96.99%. The recognition performance of the stop line is relatively poor. It can be seen from the confusion matrix that the stop line is mainly misidentified as a road surface. In terms of inference speed, the improved BiSeNet’s inference speed on Pegasus is about 31 fps, which is lower than the original BiSeNet but already meets our real-time requirements. An example of image semantic segmentation by the improved BiSeNet network is shown in Figure 3.

## 4. Semantic SLAM

After semantic segmentation, each pixel in the image is labeled with semantic tags. Then the dynamic vehicles and other outliers in the image can be filtered in the process of tracking in the dynamic environment. The meaning of the static features can be associated with points after visual SLAM mapping.

The front-end of SLAM is also called visual odometer (VO), which tracks the camera’s position and pose through the geometric relationship between multiple views. Because semi-direct VO (SVO) can combine the success-factors of feature-based methods (tracking many features) with the accuracy and speed of direct methods, it is adopted as the front end of our Visual SLAM system. As a mature and open-source method, the detailed implementation of SVO can be found in [17].

With the continuous increase of images and the continuous operation of the SLAM system, the observation error of each frame will accumulate to the next. Thus, the measurement error will continue to accumulate. Therefore, it is very important for the SLAM system to optimize trajectory on the back-end. BA (bundle adjustment) [23,24] based on graph optimization is commonly used for global optimization. The open-source graph optimization library g2o (general graph optimization) that contains the implementation of BA is utilized in this process.

When we know the camera pose from the motion estimation, the depth at a single pixel can be estimated from multiple observations by means of a recursive Bayesian depth filter [17]. From the pixel with highest correlation in the epipolar line, the depth measurement is triangulated to update the depth filter. For forward motions, it is beneficial to update the depth filters with the previous frame. While in the older version of SVO [25], the depth filter is only updated with newer frames, which works well for down-looking cameras in micro aerial vehicle applications. In fact, whether it is SVO or feature-based SLAM, such as OBR-SLAM2, triangulation is the most basic depth estimation method. Through the triangulation method, all the keyframe points can be transformed into a unified coordinate system through the corresponding perspective transformation matrix, so as to generate the point cloud map.

## 5. Vectorization and Matching with the HD Map

**Figure 4:**
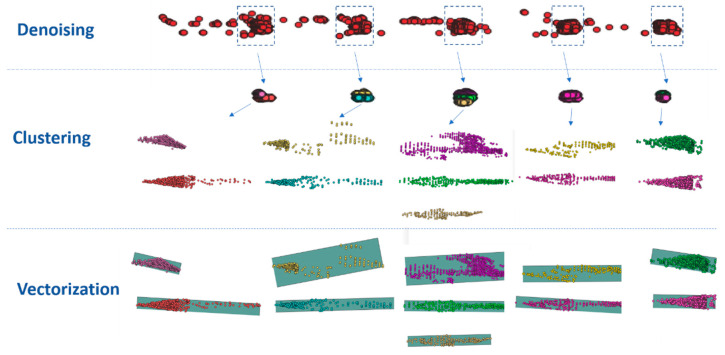
Vectorization of semantic point cloud data (taking arrows as an example).

**Figure 5:**
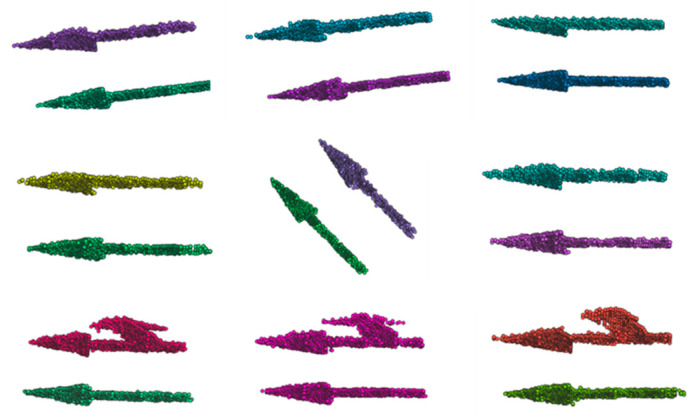
Examples of clustering of semantic point cloud.

The vectorization of semantic point cloud data consists of three steps as shown in Figure 4. The first step is denoising. As the pixel accuracy of semantic segmentation is not 100%, the noise generated needs to be filtered out. After SLAM processing, the semantic point cloud is attached to the spatial position. Thus, the Euclidean distance measure can be used for KD-Tree construction to find n points near one point and then discard the points with long distance [26]. The second is clustering. The Euclidean cluster extraction algorithm is utilized to cluster the denoised point cloud data for every object. Some examples of clustering are shown in Figure 5. We can see that the performance is acceptable. Finally, vectorization is implemented. The minimum bounding box is calculated for the surface element as its geometry. An RANSAC algorithm [27] is used for curve fitting for line elements. These algorithms are not the focus of this article, so they are not described in detail. In the process of implementation, an open-source Point Cloud Library (PCL) is used directly, which provides a lot of general point-cloud-related algorithms and efficient data structures.

After denoising, clustering, and vectorization, the vectorized results are generated. For lane dividers, line-to-line matching is needed. The matching degree is normally equivalent to the similarity calculated through distance and angle. For polygon elements like arrows, the matching degree is evaluated by the proportion of the area covered. The matching between the vectorized polygon and the HD map is illustrated by an experiment conducted on the Fifth Ring Road in Beijing.

**Figure 6:**
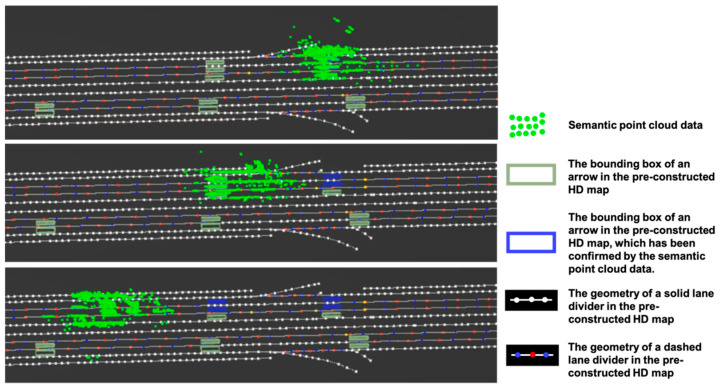
Confirmation of existence for map elements.

**Figure 7:**
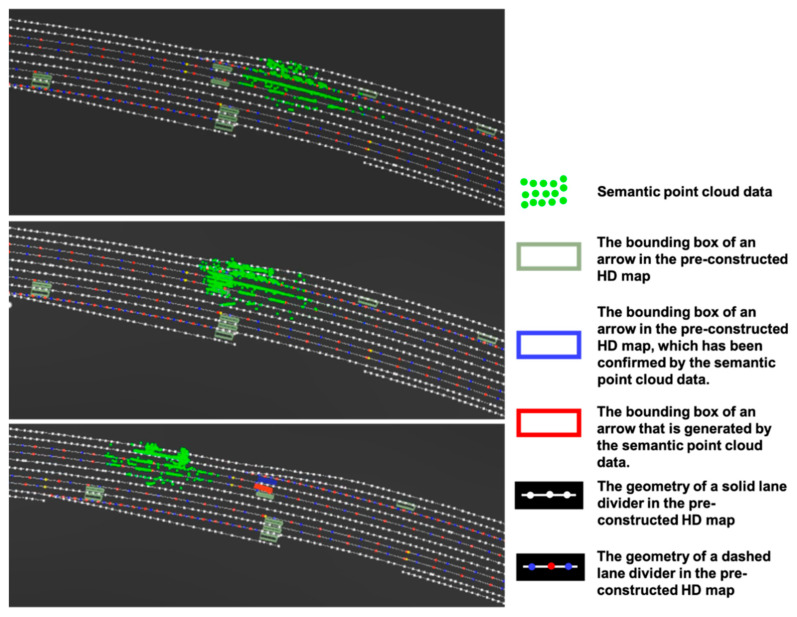
Generation of new map features from semantic point cloud.

As shown in Figure 6 and Figure 7, the green point group is the semantic point cloud data after semantic visual SLAM. The green rectangle represents the bounding box of an arrow in the pre-constructed HD map. When the car is moving, the semantic point cloud continues to grow. The vectorized results extracted from the semantic point cloud can be used to either confirm the existence of map features or generate new ones when new map features appear. In order to do the experiment, some arrows were deleted from the pre-constructed HD map.

In Figure 6, when the vehicle ran through a lane, the semantic point cloud is used to confirm the existence of arrows on the ground. It is indicated by a change of color from green to blue as shown in the middle and lower parts of Figure 6.

In Figure 7, the semantic point cloud is used to add the missing arrows, which is indicated by the new addition of red color as shown in the lower part of Figure 7.

**Figure 8:**
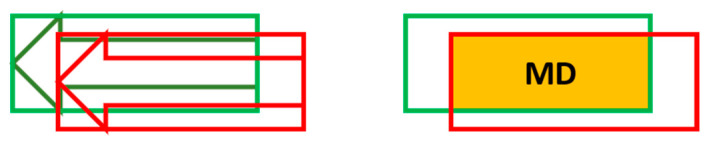
The case that bounding boxes of the map element and extracted feature cover each other.

Obviously, there is a difference in precision between the extracted features from semantic point cloud data and the map features. In order to find the corresponding map features, the matching degree between these two should be calculated. As shown in Figure 8, the green rectangle represents the bounding box of a polygon map element (e.g., an arrow), and the red represents the extracted element. The yellow part of their overlap is the area that is really matched.

The definition and calculation formula of the matching degree is as follows. That is, the matching degree is equal to the area of the overlapping divided by the area of the map element bounding box.

(8) $$\mathrm{MD}=\frac{\mathrm{Area}\left(\mathrm{Overlap}\right)}{\mathrm{Area}\left(\mathrm{Map}\right)}$$

**Figure 9:**
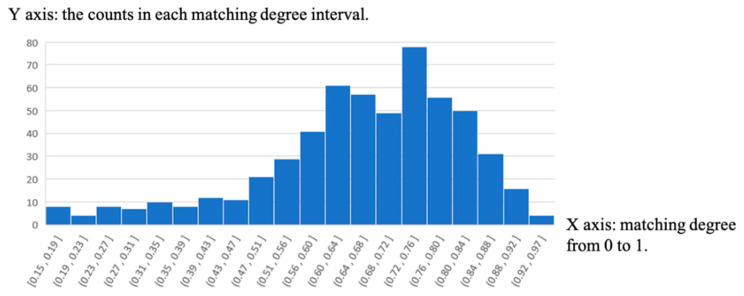
Statistical results of matching degree.

**Figure 10:**
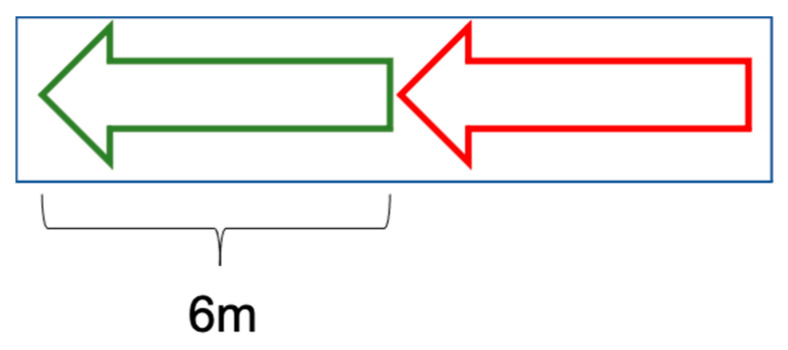
The case that bounding boxes of the map element and extracted feature don’t cover each other.

The matching degree can reflect the accuracy of the semantic point cloud data. The statistical distribution of the matching degree is shown in Figure 9. The X axis represents the matching degree. The matching degrees from 0 to 1 are divided into intervals according to a fixed range 0.05. The Y axis represents the counts in each matching degree interval. As we can see, no matching degree is between 0 and 10%. Actually, the minimum matching degree is 14.6% if matching is successful. This can indicate that features that are not matched should not be included in the map data. In other words, the case shown in Figure 10 is unlikely to happen. By the way, the length of arrows is 6 m. This conclusion is crucial to the application of crowdsourcing data.

Regarding the data volume of the semantic point cloud, the average data volume per second is 1126 KB and the average data volume per kilometer is 56,704 KB. After vectorization and matching with the HD map, the difference data is identified, which has a much smaller amount of data for transmission. The amount of the difference data depends on the changes between the old HD map and the real-time reality. The more changes, the greater the amount of the different data. In our experiment, the average amount of the different data is 126 KB per kilometer, which is much less than the amount of the whole data.

In terms of accuracy and the amount of transmitted data, the results show that the sensors and methods proposed in this paper are effective for crowdsourcing update.

## 6. Conclusions

With the unlimited range of environment perception and other advantages, the HD map is widely considered to be an essential part of autonomous driving. However, due to the complex and expensive data collection and production of HD maps, continuous maintenance and real-time updates have become a huge challenge. With the development and maturity of autonomous driving technology, more and more vehicles are equipped with a variety of advanced sensors and a powerful computing platform. Therefore, the crowdsourcing update of HD maps based on mid-to-high-end sensors is becoming more feasible.

In this context, this article uses a mature commercial GNSS/IMU integrated navigation device, an industrial camera, and NVIDIA Pegasus with GPU (Graphics Processing Unit) to launch the research. The main method is to perform real-time semantic segmentation of images based on the improved BiSeNet network and then fuse the results with visual SLAM to obtain semantic point cloud data. After denoising, clustering, and vectorization, the vectorized results are extracted from the semantic point cloud data and then matched with a pre-constructed HD map. The map elements that have not changed can be confirmed and the elements that have changes can be detected. In the experiment, the existence of arrows in the HD map is confirmed and new arrows are generated. In summary, real-time HD map change detection is realized and validated, which also demonstrates the feasibility and significant value of crowdsourcing update for HD maps.

## Figures and Tables

**Table 1:** Performance of improved BiSeNet network on Kuandeng verification dataset. IOU: Intersection over Union.

ID	Type	Recall	Pixel Accuracy	IOU
1	Traffic sign	94.40%	97.29%	91.98%
2	Pole	82.15%	90.05%	75.31%
3	Vehicles and other objects on the road	97.97%	98.97%	96.99%
4	Lane divider-white	92.42%	96.81%	89.69%
5	Lane-divider-yellow	77.32%	84.57%	67.76%
6	Speed bump	88.17%	93.42%	83.02%
7	Road surface	99.41%	98.89%	98.31%
8	Crosswalk	86.60%	97.79%	84.93%
9	Gore	94.90%	94.11%	89.57%
10	Text and symbol on the road	90.16%	93.40%	84.76%
11	Curb	86.45%	90.11%	78.96%
12	Others (Sky\Trees)	99.61%	99.37%	98.99%
13	Left road boundary	82.99%	91.94%	77.36%
14	Right road boundary	90.26%	93.14%	84.63%
15	Stop line	53.52%	94.82%	52.00%
16	Dedicated lane dividers	94.79%	92.55%	88.07%
Average		88.20%	94.20%	83.90%

## Data Availability

Publicly available datasets were analyzed in this study. This data can be found here: https://www.cityscapes-dataset.com (accessed on 1 April 2021).
